# Antimicrobial and antibiofilm activity of selected flavonoids against pathogenic *Leptospira* species

**DOI:** 10.1007/s00203-026-05067-2

**Published:** 2026-07-23

**Authors:** Walter Lilenbaum, Ignacio Gutiérrez-Del-Río, Julia Mendes, Lucas F. L. Correia, Javier Fernández, Felipe Lombó

**Affiliations:** 1 Laboratory of Veterinary Bacteriology, Biomedical Institute, Federal Fluminense University, Niterói, Rio de Janeiro, Brazil; 2 Biotechnology in Nutraceuticals and Bioactive Compounds-BIONUC, Department of Functional Biology, University of Oviedo, IUOPA (Instituto Universitario de Oncología del Principado de Asturias) and ISPA (Instituto de Investigaciones Sanitarias del Principado de Asturias), Oviedo, Spain; 3 Veterinary School, Federal Fluminense University, Niterói, Rio de Janeiro, Brazil

**Keywords:** Antimicrobial, Biofilm, Leptospirosis, Flavonoid, Quercetin

## Abstract

Leptospirosis is a widespread zoonotic disease where biofilm formation significantly hinders treatment, contributing to chronic infections. This preliminary study evaluated the antimicrobial and antibiofilm properties of four flavonoids—apigenin, xanthohumol, quercetin, and luteolin—against *Leptospira santarosai* (sv. Guaricura), *L. interrogans* (sv. Copenhageni and Hardjoprajitno), and *L. borgpetersenii* (sv. Hardjobovis). Using microdilution methods, quercetin and apigenin exhibited superior antimicrobial activity, reaching MICs of ≤ 0.12 µg/mL for *L. santarosai* sv. Guaricura. In antibiofilm assays, quercetin was the most effective compound, successfully disrupting biofilm at minimal biofilm disruptive concentrations (MBDC) as low as < 0.0012 µg/µL for *L. santarosai* sv. Guaricura and for *L. interrogans* sv. Copenhageni and Hardjoprajitno. These findings highlight the potential therapeutic candidates, requiring further evaluation, for managing both acute and persistent forms of leptospirosis, either alone or as adjuvants to current antibiotic therapies. Further in vivo studies are warranted to confirm their therapeutic safety and clinical efficacy.

## Introduction

Leptospirosis is one of the most important and widespread bacterial diseases worldwide, with a higher prevalence in tropical and subtropical regions. It is caused by Gram-negative spirochetes belonging to the genus *Leptospira*, which are capable of causing acute and severe disease in humans, clinically known as Weil’s disease (Karpagam et al., 2020). Based on genotypic classification, the genus *Leptospira* comprises pathogenic, saprophytic, and intermediate species, which are currently organized into four major phylogenetic subgroups designated as P1 (n = 17), P2 (n = 21), S1 (n = 21), and S2 (n = 5) (Vincent et al. 2019; Matiz-González et al. 2024). Although *L. interrogans* is the most frequently associated species with human and animal infections, several other pathogenic species have also been reported, including *L. borgpetersenii*, *L. kirschneri*, *L. mayottensis*, *L. noguchii*, *L. santarosai*, *L. weilii*, and *L. alexanderi* (Chinchilla et al. 2023). In addition to genotypic classification, serological classification remains widely used in epidemiological studies and is based on variations in the composition of surface lipopolysaccharides (LPS), which define the different serovars (sv.) of *Leptospira* (Mejía et al. 2022).

The primary route of leptospirosis infection is indirect, occurring through contact of mucous membranes or abraded skin with water, soil, or food contaminated by the urine of infected animals. Infection risk increases in humid environments and during flooding events, which promote leptospiral survival and dissemination, particularly in urban areas with poor sanitation and high rodent densities (Bierque et al. 2020). In humans, leptospirosis ranges from mild febrile illness to severe disease characterized by acute renal failure, jaundice, pulmonary hemorrhage, and multiple organ dysfunction (Sykes et al. 2022). Its increasing global incidence has been associated with climate-related factors, such as global warming and flooding, as well as human activities including ecotourism and military operations in tropical regions (Dharmashekar et al. 2023). In dogs and horses, the disease typically presents as an acute systemic infection, whereas in cattle and swine it is usually chronic, causing reproductive disorders and significant economic losses (Sykes et al. 2023; Aymeé et al. 2024).

Leptospirosis is a zoonosis strongly influenced by environmental and interspecies interactions, making it a representative model for the One Health approach (Sykes et al. 2022). Among pathogenic species, *L. interrogans* is the most extensively studied and is commonly associated with human and cattle infections by the Icterohaemorrhagiae and Sejroe serogroups, respectively. However, other pathogenic species have gained increasing epidemiological relevance, particularly *L. borgpetersenii* (sv. Hardjobovis) and *L. santarosai*. Recent studies have shown that *L. santarosai* is widely distributed throughout the Americas and infects humans, livestock, companion animals, and wildlife, being increasingly associated with bovine reproductive disease (Chinchilla et al. 2023; Aymée et al. 2024; Pélaez-Sánchez et al. 2026). In humans, *L. santarosai* has been associated with the full clinical spectrum of leptospirosis, ranging from mild febrile illness to severe manifestations, including Weil's syndrome, pulmonary hemorrhage, and neuroleptospirosis (Pélaez-Sánchez et al. 2026). Despite its growing epidemiological relevance, important aspects of its biology, persistence mechanisms, and pathogenicity remain poorly understood (Pélaez-Sánchez et al. 2026).

Biofilm formation represents one of the main mechanisms responsible for the environmental persistence and host colonization of *Leptospira*. These structures consist of microbial communities embedded in an extracellular matrix composed of proteins, polysaccharides, and extracellular DNA, which protects bacteria from environmental stress, host immune responses, and antimicrobial exposure (Luo et al. 2024). This adaptive lifestyle contributes to bacterial persistence, antimicrobial tolerance, and chronic renal colonization, increasing the likelihood of therapeutic failure (Flemming and Wuertz 2019; Dias et al. 2025). The ability to form biofilms has been demonstrated in several *Leptospira* species under both in vitro and in vivo conditions (Ristow et al. 2008; Meganathan et al. 2022). Recent studies have implicated *L. interrogans* biofilms in the pathogenesis of equine recurrent uveitis (Wollanke et al. 2022), renal colonization in rodents (Santos et al. 2021), and acute systemic disease in dogs (de Carvalho et al. 2023; Dias et al. 2025), highlighting their importance in the epidemiology, persistence, and clinical outcomes of leptospiral infections.

Treatment of leptospirosis remains challenging in both humans and animals (Mendes et al. 2025). Traditionally, penicillin and doxycycline are the drugs of choice for humans and dogs, while streptomycin is widely recommended for ruminants (Sykes et al. 2023; Win et al. 2024). Although most clinical isolates remain susceptible to antibiotics commonly used to treat leptospirosis, including β-lactams, tetracyclines, and macrolides, genomic analyses have revealed the presence of several apparent determinants of antimicrobial resistance (AMR), such as efflux pumps and aminoglycoside-modifying enzymes, which may contribute to reduced susceptibility or adaptive responses under specific environmental conditions (Liegeon et al. 2018; Correia et al. 2019; Petakh & Kamyshnyi 2024; Pineda et al. 2024). However, these findings are largely based on in silico analyses and should not be interpreted as direct evidence of phenotypic resistance (Petakh & Kamyshnyi 2024). Nevertheless, the growing recognition of biofilm formation, bacterial persistence, and potential AMR-related mechanisms in *Leptospira* highlights the need for novel therapeutic approaches capable of targeting both acute infection and long-term bacterial survival. In this context, natural and synthetic compounds have attracted increasing attention due to their antimicrobial, antibiofilm, and synergistic potential with conventional antibiotics (Dias et al. 2025).

Among these compounds, flavonoids are particularly promising because of their well-documented antimicrobial, antioxidant, and anti-inflammatory properties (Ji et al. 2024; Pathak & Mazumder 2024). Previous studies have demonstrated the activity of flavonoids such as quercetin and apigenin against several bacterial pathogens, including *Leptospira interrogans* (Cushnie & Lamb 2011; Rajalakshmi et al. 2025). In particular, quercetin has shown antibacterial and antibiofilm activity, including the modulation of virulence- and adhesion-associated genes in *L. interrogans* (Rajalakshmi et al. 2025). These findings suggest that flavonoids may act through complementary antibacterial and antibiofilm mechanisms, making them attractive candidates for both veterinary and human applications. However, the activity of flavonoids remains poorly explored against epidemiologically important species such as *L. santarosai*, despite the increasing recognition of this species as an important pathogen of humans and livestock and the major knowledge gaps regarding its persistence and biology (Pélaez-Sánchez et al. 2026). Likewise, information regarding the susceptibility of *L. borgpetersenii*, one of the principal agents of bovine leptospirosis worldwide, remains scarce (Aymée et al. 2024). Therefore, this preliminary study evaluated the antimicrobial and antibiofilm activities of flavonoids against four pathogenic *Leptospira* strains: *L. santarosai* (sv. Guaricura), *L. interrogans* (sv. Copenhageni and Hardjoprajitno), and *L. borgpetersenii* (sv. Hardjobovis).

## Materials and methods

### Strains

The chosen strains for this project are *L. santarosai* sv.Guaricura, *L. interrogan*s serovars Copenhageni and Hardjoprajitno, and *Leptospira borgpetersenii* sv. Hardjobovis, all from the collection of the Veterinary Bacteriology Laboratory of the Fluminense Federal University. The strains were characterized and confirmed following the protocol described by Di Azevedo et al. (2025). For each strain, the accession number, host source, year of isolation, and geographic origin were recorded (Table 1). Before the experiments, all strains were reisolated after in vivo passage in Golden Syrian hamsters (*Mesocricetus auratus*) to restore virulence and were used at the second in vitro passage. Experiments were conducted in a BSL-2 facility. The strains were thawed and maintained in Ellinghausen–McCullough–Johnson–Harris medium (EMJH–BD Difco™, Franklin Lakes, NJ, USA) at 30 °C for seven days and were free of contamination or autoagglutination.

**Table 1:** Identification, origin, and host of pathogenic *Leptospira* strains used in this study

Species	Serovar	Accession no	Host	Year	Origin
*L. santarosai*	Guaricura	JAUOTG000000000	Bovine	2013	Brazil
*L. interrogan*s	Copenhageni	AE016823	Human	1996	Brazil
*L. interrogan*s	Hardjoprajitno	CP012604	Bovine	2016	Brazil
*L. borgpetersenii*	Hardjobovis	ANMU00000000	Bovine	2013	Netherlands

### Compounds and concentration ranges

A total of four commercial flavonoids (> 90% purity) were evaluated: apigenin (Merck, ref. 10,798, CAS number 8002–66-2), xanthohumol (Merck, ref. 01130595, CAS number 6754–58-1), quercetin (Merck, ref. Q4951, CAS number 6151–25-3), and luteolin (Merck, ref. L9283, CAS number 491–70-3), plus one reference drug, streptomycin (Sigma-Aldrich, ref. s6501, CAS number 57–92-1), used as a positive control, totaling five tested drugs. Stock solutions of the flavonoids (1.25 mg/mL) were prepared in 10% methanol and then the working solutions made in EMJH medium to yield final concentrations ranging from 250 µg/mL (final methanol concentration 2% in the microtiter well) to 0.12 µg/mL (final methanol concentration 0.0009%). Blank controls were performed with EMJH medium containing the same methanol concentrations.

### Minimum inhibitory concentration (MIC)

Broth microdilution assays were performed in 96-well flat-bottom microtiter plates as previously described (Murray et al., 2004; de Carvalho et al. 2023). Each plate included positive controls (bacteria without antimicrobial compounds), negative controls (medium only), and 12 twofold serial dilutions of each biocompound prepared in EMJH medium, resulting in final concentrations ranging from 250 to 0.12 µg/mL. The leptospiral inoculum was adjusted to 10⁶ leptospires/mL, and the final volume in each well was 200 µL. All experiments were performed in duplicate. Plates were incubated at 30 °C for five days in a sterile distilled water-saturated atmosphere to minimize evaporation. After incubation, bacterial growth was independently evaluated by spectrophotometric measurement at 492 nm using a BioTek ELISA microplate reader and by dark-field microscopy (DFM). Spectrophotometric readings (OD492) were used as an indirect indicator of leptospiral growth and culture turbidity. MIC determination was based on spectrophotometric evaluation and subsequently confirmed by dark-field microscopy (DFM), which was considered the definitive method for assessing leptospiral viability through the observation of motility, morphology, and growth. Accordingly, the minimal inhibitory concentration (MIC) was defined as the lowest concentration showing no detectable increase in turbidity relative to the negative control and no evidence of viable leptospires under DFM.

### Biofilm inhibition assay

Antibiofilm activity was evaluated in all four pathogenic *Leptospira* strains after 21 days of incubation, when mature biofilm formation was observed under dark-field microscopy (DFM). Biofilm maturity was defined by the presence of dense adherent bacterial aggregates and extracellular matrix structures attached to the well surface, as previously described for leptospiral biofilms (Ristow et al. 2008; Thibeaux et al. 2020). Following visual confirmation of mature biofilm on day 21, the four compounds were added in a two-fold serial dilution scheme (10 dilutions ranging from 62.5 to 0.0012 μg/mL), prepared from an initial stock concentration of 1,250 μg/mL in EMJH medium. Plates were further incubated for five days at 30 °C and evaluated on day 26. Biofilm disruption was assessed qualitatively by DFM. No quantitative biofilm assay was performed. The minimal biofilm disruptive concentration (MBDC) was defined as the lowest concentration capable of causing visible biofilm disorganization or disruption when compared with untreated control wells.

## Results

The four biocompounds (besides the positive control of the antibiotic streptomycin) were tested for their in vitro anti-leptospiral activity using the microdilution method and the dark field microscopic technique against the chosen strain. It was observed that during the microdilution test, all of them showed some inhibitory activity, at concentrations ranging from ≤ 0.12 µg/mL for quercetin and apigenin against *L. santarosai* sv Guaricura, up to 1.95 µg/mL for luteolin against *L. santarosai* sv Guaricura, *L. interrogans* sv Hardjoprajitno, and *L. borgpetersenii* sv Hardjobovis (Table 2).

**Table 2:** Inhibitory activity of Flavonoids against *L. santarosai* serovar Guaricura, *L. interrogan*s serovars Copenhageni and Hardjoprajitno, and *Leptospira borgpetersenii* serovar Hardjobovis. Data from two independent replicate experiments

Strain	Compound	MIC^1^
*L. santarosai* sv. Guaricura	Streptomycin	0.24
	Quercetin	≤ 0.12
	Apigenin	≤ 0.12
	Luteolin	1.95
	Xanthohumol	0.97
*L. interrogan*s sv. Copenhageni	Streptomycin	0.12
	Quercetin	0.24
	Apigenin	0.24
	Luteolin	0.97
	Xanthohumol	0.97
*L. interrogan*s sv. Hardjoprajitno	Streptomycin	0.97
	Quercetin	0.24
	Apigenin	0.24
	Luteolin	1.95
	Xanthohumol	0.97
*L. borgpetersenii* sv. Hardjobovis	Streptomycin	0.97
	Quercetin	0.24
	Apigenin	0.24
	Luteolin	1.95
	Xanthohumol	0.97

^1^Concentration at µg/mL

When compared to the standard drug streptomycin, we could notice that, except for sv Copenhageni, MICs of quercetin and apigenin were similar or even lower than the standard drug, particularly against *L. santarosai* sv Guaricura. Moreover, those two flavonoids presented the expected and desired antibacterial effect, in contrast to luteolin and Xanthohumol.

**Fig. 1:**
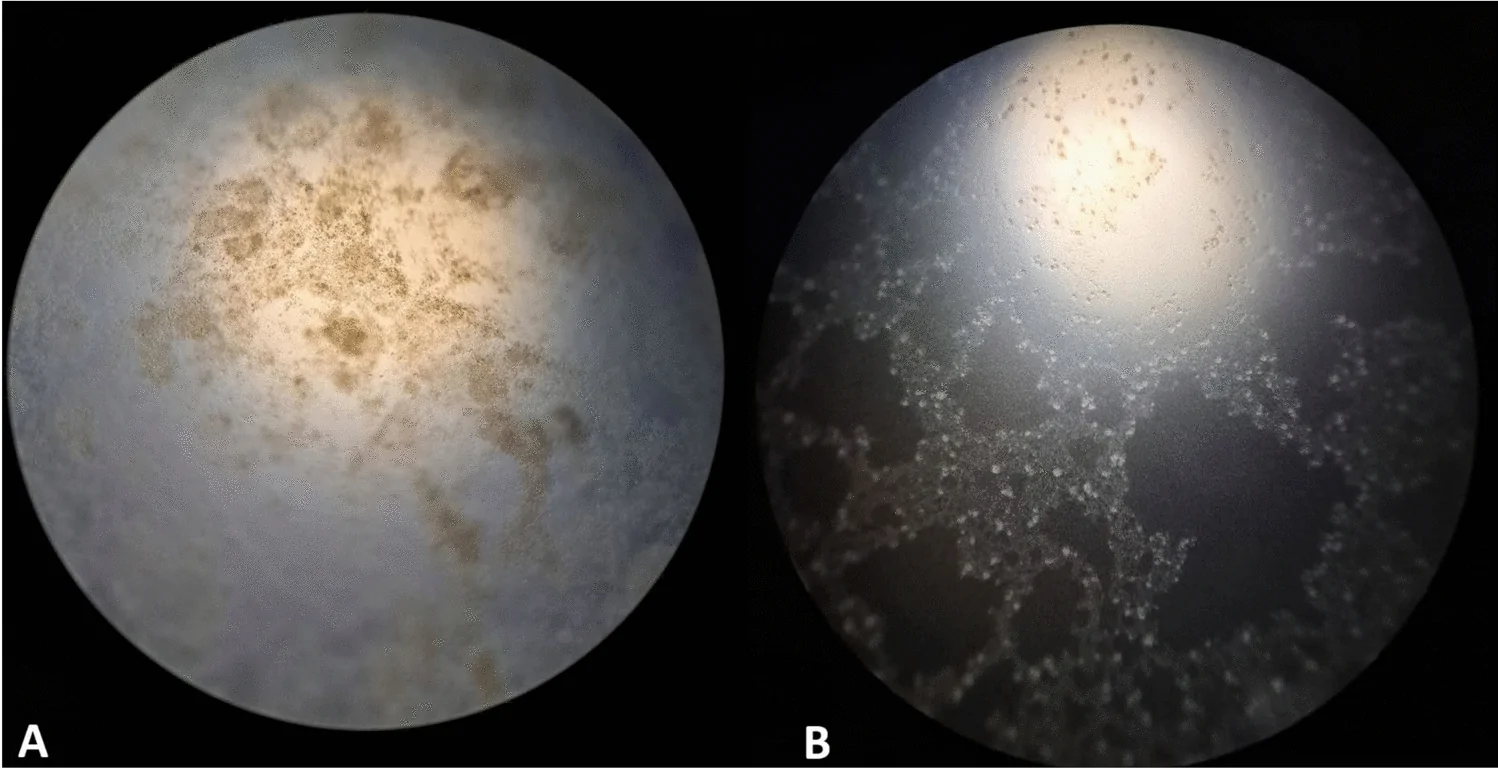
Dark-field microscopy images (600x) of microtiter wells corresponding to: A) a *L. santarosai* biofilm positive control well at 21 days; B) quercetin treatment (5 days) at 0.0012 µg/mL final concentration

In relation to the antibiofilm activity, all four compounds were able to disrupt the biofilm for the four leptospiral strains (except for luteolin in *L. santarosai* sv Guaricura) in different MBDCs (Table 3). Best results were observed for quercetin, which disrupted biofilm on MBDCs as low as < 0.0012 µg/mL for *L*. *santarosai* sv Guaricura, and *L. interrogans* sv Copenhageni and Hardjoprajitno. Microscopic examination confirmed decreased biofilm density and structural disorganization in treated groups compared with controls (Fig. 1).

**Table 3:** Antibiofilm activity of Flavonoids against *L. santarosai* sevovar Guaricura, *L. interrogan*s serovars Copenhageni and Hardjoprajitno, and *L. borgpetersenii* serovar Hardjobovis

Compound	MBDC *L. santarosai* (sv. Guaricura)	MBDC *L. interrogans* *(*sv. Hardjoprajitno)	MBDC *L. interrogans* (sv. Copenhageni)	MBDC *L. borgpetersenii* (sv. Hardjobovis)
Quercetin	< 0.0012 µg/mL	< 0.0012 µg/mL	< 0.0012 µg/mL	0.97 µg/mL
Apigenin	0.005 µg/mL	0.97 µg/mL	0.97 µg/mL	1.95 µg/mL
Luteolin	-	0.48 µg/mL	0.48 µg/mL	0.48 µg/mL
Xanthohumol	0.08 µg/mL	0.48 µg/mL	0.48 µg/mL	1.95 µg/mL

## Discussion

This is a preliminary study, and limitations should be considered when interpreting the results. First, biofilm disruption was evaluated qualitatively by dark-field microscopy, and no quantitative biofilm measurements were performed. In addition, cytotoxicity assays were not conducted, preventing conclusions regarding the safety profile of the compounds evaluated. The study also relied on technical duplicates and did not include independent biological replicates, precluding inferential statistical analyses. Finally, quercetin and apigenin exhibited MIC values at or below the lowest concentration tested (≤ 0.12 µg/mL), indicating that the exact MIC could not be determined and was therefore reported as ≤ 0.12 µg/mL. Despite these limitations, the findings suggest that selected flavonoids present both antimicrobial and antibiofilm activities against pathogenic *Leptospira* spp., supporting their further investigation. Among the compounds evaluated, quercetin and apigenin consistently exhibited the most encouraging outcomes, indicated by the lowest MIC values and the strongest antibiofilm effects under the experimental conditions tested.

Interestingly, quercetin and apigenin exhibited MIC values at or below the lowest concentration tested (≤ 0.12 µg/mL), suggesting high susceptibility among the strains evaluated. Because the lower limit of the dilution series was reached, the exact MIC could not be determined. Although these values are lower than those commonly reported for flavonoids against many bacterial pathogens, further studies including expanded dilution ranges, biological replicates, and complementary viability assays are necessary to confirm their reproducibility and biological significance.

The dual activity observed aligns with the findings reported by Rajalakshmi et al. (2025), who demonstrated that quercetin inhibits both growth and biofilm formation in *Leptospira interrogans*. Our results extend these observations to *L. santarosai,* a species of major veterinary importance. Flavonoids are widely distributed polyphenolic compounds that have demonstrated antimicrobial activity against a broad range of bacterial pathogens. Previous studies suggest that their antibacterial effects may involve multiple mechanisms, including membrane destabilization, inhibition of efflux pumps, interference with microbial enzymes, and modulation of gene expression (Osman et al. 2024; Pathak & Mazumder 2024; Zhang et al. 2025). Although these mechanisms were not investigated in the present study, they may contribute to the antimicrobial activity observed for quercetin and apigenin.

Regarding the antibiofilm activity, the most encouraging results were observed for quercetin, which disrupted biofilms at concentrations lower than 0.0012 μg/mL in three out of the four strains evaluated (Table 3). These findings extend previous observations on the antibiofilm potential of flavonoids and reinforce the relevance of exploring natural compounds as alternative approaches for controlling leptospiral biofilms.

The antibiofilm activity observed in the present study is consistent with reports describing the ability of flavonoids to interfere with biofilm formation and stability. Rajalakshmi et al. (2025) demonstrated that quercetin inhibits both growth and biofilm formation in *Leptospira interrogans*, supporting the findings reported here. Similar effects have been reported in other bacterial species, where quercetin-based formulations reduced biofilm biomass and promoted structural disruption of mature biofilms (Luo et al. 2023). Additionally, plant-derived polyphenolic compounds have been shown to inhibit biofilm formation and attenuate virulence-associated traits in oral pathogens such as *Prevotella intermedia* and *Porphyromonas gingivalis* (Peeran et al. 2024). Although the precise mechanisms in leptospires remain unclear, flavonoids are known to affect multiple targets involved in biofilm development and persistence, including membrane integrity, extracellular polymeric matrix stability, and cellular processes required for biofilm maturation. The strong antibiofilm activity observed for quercetin may be partially related to its hydroxylation pattern, particularly at the C3 and C3' positions, which has been associated with enhanced interactions with bacterial envelope components and greater antimicrobial activity in structure–activity relationship studies (Pathak & Mazumder 2024).

The relevance of these findings is reinforced by the growing evidence that biofilm formation contributes to leptospiral persistence and reduced susceptibility to antimicrobial compounds. Recently, Dias et al. (2025) demonstrated that *Leptospira interrogans* biofilms exhibit substantially greater tolerance to antibiotics than their planktonic counterparts. Therefore, compounds capable of disrupting established biofilms may represent valuable adjuncts to conventional antimicrobial therapy and contribute to overcoming biofilm-associated persistence in leptospiral infections.

Leptospirosis remains an important zoonosis affecting both humans and animals worldwide. In humans, infection is frequently associated with exposure to environments contaminated with urine from infected reservoir hosts, particularly rodents, and may range from mild disease to severe, potentially fatal forms requiring intensive medical care (Linhares et al. 2024). In livestock, leptospirosis is commonly associated with reproductive disorders, including abortion, infertility, and reduced productivity, resulting in substantial economic losses. In both scenarios, the identification of safe and effective alternative compounds capable of complementing current therapeutic approaches remains an important area of investigation.

One of the most interesting findings of this study was the demonstration of biofilm formation by *L. santarosai*. This species has been increasingly recognized as an important pathogen in both human and animal leptospirosis throughout the Americas, and has been frequently associated with bovine reproductive infections (Chinchilla et al. 2023; Aymée et al. 2024). Recent systematic evidence has highlighted that important knowledge gaps still exist regarding the biology, pathogenicity, and persistence mechanisms of *L. santarosai*, with no previous reports describing biofilm formation in this species (Pélaez-Sánchez et al. 2026). Therefore, to the best of our knowledge, the present study provides the first evidence of in vitro biofilm formation by *L. santarosai* and the first description of biofilm production by a bovine-origin leptospiral strain. These findings expand the current understanding of this emerging pathogen and provide a basis for future studies investigating the role of biofilms in environmental persistence, host colonization, pathogenesis, and treatment response.

Beyond its potential ecological role, biofilm formation has important therapeutic implications. Biofilm-associated leptospires exhibit increased tolerance to antimicrobial compounds compared with planktonic bacteria. Therefore, understanding the mechanisms involved in biofilm formation and identifying compounds capable of disrupting these structures may contribute to the development of improved therapeutic strategies for leptospirosis. Future studies should evaluate the pharmacokinetic properties, cytotoxicity, and in vivo efficacy of these flavonoids, as well as their potential synergistic effects with conventional antimicrobial agents.

## Conclusions

In conclusion, this preliminary study demonstrated the in vitro antimicrobial and antibiofilm activities of selected flavonoids against pathogenic *Leptospira* strains, with quercetin and apigenin showing the most encouraging outcomes, demonstrated by the greatest activity among the compounds evaluated. Moreover, this study provides the first published evidence of in vitro biofilm formation by *L. santarosai*, expanding current knowledge of this emerging pathogen. Considering the increasing recognition of *L. santarosai* as an important pathogen of humans and livestock throughout the Americas, a better understanding of its biofilm biology may contribute to future studies on bacterial persistence, pathogenesis, and disease control. Although these findings highlight the potential of flavonoids as candidate compounds for further investigation, additional studies evaluating their cytotoxicity, pharmacokinetic properties, efficacy in vivo, and possible synergistic interactions with conventional antimicrobials are required before any therapeutic application can be proposed.

## Data Availability

No datasets were generated or analysed during the current study.
